# Manure and tillage use in remediation of eroded land and impacts on soil chemical properties

**DOI:** 10.1371/journal.pone.0175533

**Published:** 2017-04-27

**Authors:** Maysoon M. Mikha, Joseph G. Benjamin, Merle F. Vigil, David J. Poss

**Affiliations:** US Department of Agricultural, Agricultural Research Service, Central Great Plain Research Station, Akron, Colorado, United States of America; Pacific Northwest National Laboratory, UNITED STATES

## Abstract

Soil loss through wind and water erosion is an ongoing problem in semiarid regions. A thin layer of top soil loss over a hectare of cropland could be corresponding to tons of productive soil loss per hectare. The objectives of this study were to evaluate the influence of beef feedlot manure, tillage and legume grass mixtures on changes in soil quality and nutrient components. The study was initiated in 2006 on an eroded site near Akron, Colorado, on a Norka-Colby very-fine sandy loam (fine-silty, mixed, mesic, Aridic, Argiustolls). Tillage treatments were no-tillage, shallow tillage (sweeps operations with V-blade) and deep tillage (DT; moldboard plow operations). In one set of plots, DT was implemented biannually (DT-2); and in another set the DT was done once at the initiation of the experiment in 2006. Amendments consisted of beef manure and urea (46-0-0), N fertilizer. Both amendments were added at low and high rates. A control treatment, with no fertilizer or manure added, was included with no-tillage and shallow tillage only. Six years of manure addition and tillage significantly altered soil chemical properties compared with fertilizer and grass legume mixtures. Across all the tillage treatments, at the 0–30 cm depth, soil pH from 2006 to 2012, was reduced 1.8 fold with high-manure compared with high-fertilizer treatment. Soil EC, Na, and SAR increased by 2.7 fold while soil P increase by 3.5 fold with high-manure treatment compared with low-manure from 2006 to 2012 across all the tillage treatments at the surface 0–30 cm. Soil organic carbon associated with high-manure was 71% higher than low-manure and 230% higher than high-fertilizer treatments in the 0–60 cm depth. Similar patterns were observed with soil total N. Overall, manure amendments greatly improved the soil nutrient status on this eroded site. However, the legume grass mixtures showed little effect on improving soils chemical properties. The micronutrients supplied by manure improved the soil nutrient status compared with inorganic fertilizer, the grass, and the grass-legume treatments. We concluded that more than six years are needed to measure significant improvements in soil quality from specific treatments, specifically fertilizer, grasses, and grass-legume mixtures in such eroded crop land.

## Introduction

Soil erosion by wind and water, continues to be a problem in many croplands around the USA. Previously, [1] documented that even one mm of soil loss, over a hectare of cropland, is equivalent to 15 t ha^-1^ of surface soil eroded. Losing surface soil that is rich with organic matter and nutrient adversely affects crop yields [2–4] due to its exceptional characteristics in nutrient cycling, water storage, and energy transfer. The thickness of surface soil is an important factor in evaluating soil quality and sustainability [5]. Therefore, various management practices need to be implemented to replace the lost top soil because it could require more than 20 years through natural soil formation processes [1].

**Fig 1:**
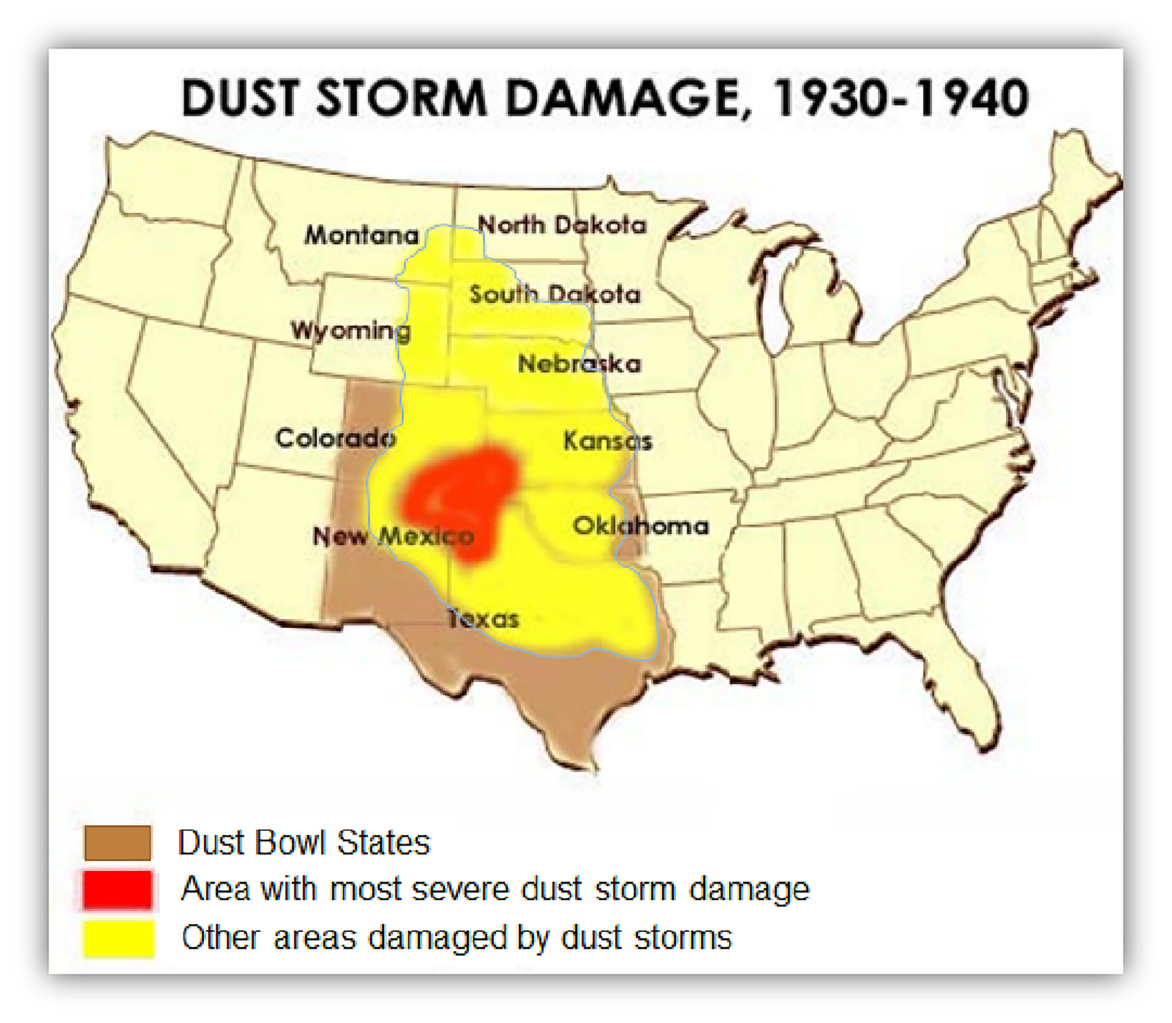
States affected by severe dust storm “Dust Bowl” that occurred during the 1930’s to 1940’s. The center of the storm was in Panhandles of Texas and Oklahoma, but its significant effect damaged the neighboring states.

Historically, some farmland in the Great Plains of North America was economically devalued as a consequence of wind and water erosion induced by drought and tillage [2–4, 6]. As the undisturbed grassland of the semiarid region of the Great Plains was converted to farmland in the early 1900’s, wind erosion and soil degradation became a critical problem [3, 6]. Prior to the 1930’s, climatic conditions were favorable for cultivation, where average or above-average precipitation in combination with good native soil fertility was favorable for wheat production [3]. With the high demand for wheat after World War I, in combination with technological improvements in farming, more grassland was converted to cropland in the semi-arid Great Plains region [3]. At the beginning of the 1930’s, the Great Plains experienced an unusually dry era that lasted till 1940. Dry, hot conditions, in combination with normally high winds and clean tillage practices, resulted in huge amounts of topsoil loss rich with organic matter and nutrients, through wind erosion, historically known as the Dust Bowl [3]. The Dust Bowl (Fig 1) centered on the Panhandles of Texas and Oklahoma and extended to New Mexico, Colorado, and Kansas.

**Fig 2:**
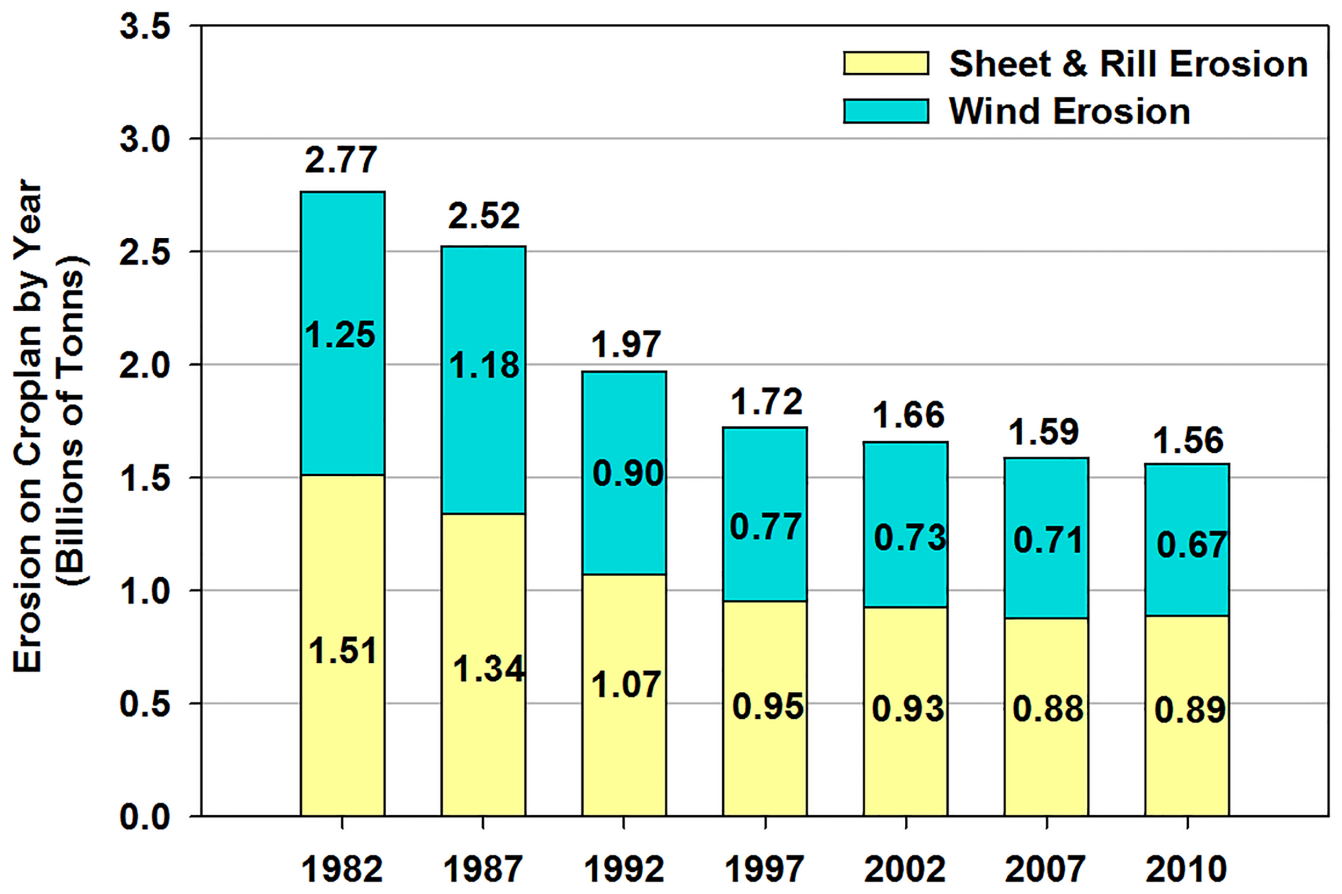
Cropland represents cultivated and non-cultivated cropland. Reduction in cropland erosion process, water and wind erosion, around the United State of America from 1982 to 2010. Figure is adapted and reconstructed into metric tons (Tonns) from data provided by U.S. Department of Agriculture, *Summary Report*: *2010 National Resources Inventory*, Natural Resources Conservation Service, September 2013. Courtesy of USDA’s Natural Resources Conservation Service (NRCS). http://www.nrcs.usda.gov/Internet/FSE_DOCUMENTS/stelprdb1167354.pdf.

During the last few decades, conservation practices with reduced tillage or no-tillage have been adopted in the Great Plains [3]. Cropland wind erosion in the U.S.A. (Fig 2) has decreased from 1.38 billion ton per year in 1982 to 0.74 billion ton year^-1^ in 2010 [7]. Specifically, in the state of Colorado, average annual wind erosion, on cropland, decreased from 30.34 t ha^-1^ in 1982 to 25.54 t ha^-1^ in 2010 [8]. Wind erosion remains a major cause of soil degradation [1, 9] in the semi-arid Great Plains, even with the recent increase in resource conservation practices [3].

Remediation/restoration of eroded cropland can be achieved by adding organic amendments (e.g. manure) to replenish SOM lost through erosion [4, 10–12]. Organic amendments also enhance microbial activity and microbial diversity [13]. Previously, [10, 14] observed an improvement in soil physical properties of eroded land with manure amendments. They reported decreased soil bulk density, increased soil water retention, and improved soil hydraulic properties. Previously, [13] reported that soil biogeochemical cycling, necessary for agroecosystem production, was improved with manure additions compared with inorganic fertilizer in eroded sites in Colorado, Kansas, and Kentucky. Six years of annual manure applications on eroded land in central Kansas, improved soil nutrient dynamics, increased SOC, and improved cropland production [12, 15]. Soil properties, such as reduced soil pH, enhanced SOC, increased N and P availability, and elevated electrical conductivity (EC), were associated with increased manure application rates [12]. Furthermore, converting eroded cropland to grass land could be a management option to reduce land degradation, with continuous soil disturbance due to cultivation, and enhancing soil organic matter accumulation. In the Great Plains Region of our study site, [16] reported that perennial grasses treatment increased soil organic C and N to a level similar to native prairie after 4 years of reestablishment.

Most of the reported studies dealing with restoration of degraded cropland have been conducted in the humid Central and Eastern regions of the USA on neutral to acid soils [10, 17]. The weather patterns of the West Central Great Plains region tend to by relatively dry, with calcareous soils and low SOM. It may be unrealistic to assume that remediation practices suitable to humid regions are suitable for semiarid regions. In fact, the devastating Dust Bowl that occurred in the 1930’s was partially due to the adoption of humid-region management practices in a semiarid environment [3]. The primary tillage practice at that time was moldboard plowing and disking. Using these tillage practices on medium to fine textured soils of the wheat-fallow cropping system, in combination with low precipitation, all contributed to the notorious 1930’s Dust Bowl in the Great Plains region ([3, 6, 18]. Unlike the acid soils of the Eastern or Central USA, the carbonate content in the calcareous soils of the Western USA can affect nutrient availability and solubility [19]. Much of the remediation research in the Great Plains region has been conducted on simulated erosion sites at different levels of topsoil removal and manure amendments [2, 11]. Few studies report remediation practices using organic amendments on environmentally and anthropogenically eroded sites in the Great Plains [12–13, 15].

Best management practices for the restoration of eroded, calcareous soil in the Western USA with the addition of organic amendments have not been adequately quantified. Further knowledge on the influence of organic amendments and tillage practices on nutrient dynamics and redistribution are needed to prevent cropland degradation in the semiarid west of the USA. Previous research in this region [12–13, 15] evaluated no-tillage and shallow tillage with annual beef manure addition at different rates. However, in the current study, treatments with moldboard plowing (deep tillage; approximately 27 to 36 cm depth) at different beef manure rates and frequencies were also included. The deep tillage treatments were included to evaluate the influence of the mechanical mixing of beef manure, to a deeper depth, on soil chemical composition compared with no-tillage or shallow tillage. We hypothesized that the moldboard plowing (DT) of beef manure once every six years may be as beneficial as surface annual manure application to improve nutrient dynamics in the soil profile of this eroded site. We also hypothesized that the micronutrients supplied by beef manure would enhance soil fertility of the eroded soils beyond what would be expected with just chemical addition of N and P fertilizer. The objective of this study is to evaluate changes in soil chemical properties of eroded cropland as influenced by (1) applications of solid beef manure compared to no amendment and chemical N and P: (2) frequency of manure applications; (3) tillage practices; and (4) converting the land back to permanent grassland.

## Materials and methods

### Site and treatment descriptions

The study was initiated in 2006 on an eroded farmer’s field near Akron, CO. The study lies at 40° 04’ 48.2 N latitude and 106° 06’ 55.6 W longitude approximately 1384 m above mean sea level. The soil series in this study site is Norka-Colby very fine sandy loam (fine-silty, mixed, mesic, Aridic, Argiustolls) with approximately 5% slope. Soil texture consists of 350 g kg^-1^ sand, 450 g kg^-1^ silt, and 200 g kg^-1^ clay. The 105 yr. average annual precipitation is 420 mm.

Based on the current soil profile compared with untilled Norka-Colby soils, it is estimated that this site, through time, lost approximately 17 cm of its topsoil to erosion. This is equivalent to losing the A horizon (0–10 cm) exposing the Bt horizon on which the farming operation is currently being conducted. Before the initiation of this study in 2006, this site was managed by the farmer with wheat-millet-fallow rotation. The site was annually tilled (v-blade sweep at the depth of 10 to 15 cm) to control weeds between crops. Inorganic N fertilizer was used with no manure added prior to 2006. This site was chosen for remediation/restoration purposes because of its eroded characteristics and low productivity, approximately 35–45%, relative to neighboring fields [20].

The experiment is being conducted in plots large enough to accommodate full size farm equipment. Individual experimental units are approximately 13.7 m wide by 15 m long. The cropping sequence used was typical for the region. Proso millet (*Panicum miliaceum* L.) was grown in 2007, winter forage triticale (x *Triticosecale*) was grown in 2007–2008, winter wheat (*Triticum aestivum* L.) was grown in 2008–2009, proso millet (*Panicum miliaceum* L.) was grown in 2010, corn (*Zea mays* L.) was grown in 2011and winter wheat (*Triticum aestivum* L.) was grown in 2012–2013. Due to exceptionally dry conditions, the field was summer fallowed in 2012. Detailed descriptions of crops, varieties and field operations are shown in Table 1. The study plots are arranged in randomized complete block design with four replicates for all treatments. Weeds were controlled using no-till practices primarily glyphosate, [isopropylamine salt of *N-*(phosphonomethyl) glycine], 2,4-D, and dicamba herbicides. Three tillage treatments were implemented in this study exclusively for incorporation of the manure. These tillage treatments were implemented soon after manure application. Tillage treatments consisted of (i) no-tillage (NT) where the manure was left on the soil surface and all weeds are controlled with herbicides (primarily with glyphosate, [isopropylamine salt of *N-*(phosphonomethyl) glycine]; (ii) shallow tillage (ST; that consisted of a v-blade sweep operation approximately 8–12 cm deep to incorporate the manure or fertilizer); and (iii) deep tillage with a moldboard plow (DT; fall to late summer a moldboard plowing at approximately 27 to 36 cm depth, followed by chiseling at approximately 20–25 cm depth to mix the soil and smooth the seedbed). The DT treatment was implemented in two ways. In one set of plots the DT was performed biannually, once every other year (DT-2). In the second set of the plots, the DT was performed once at the beginning of the study in fall of 2006 (DT-6).

**Table 1:** Crop description and field operation information since the initiation of the study in fall 2006 to 2013.

Year	Crop	Variety	Seeding rate	Planting	Grain harvesting
			-- kg ha^-1^ --	---------- Date ----------	
2007- s^†^	Proso millet	Early Bird	17.0	June 22, 2007	September 20, 2007
2007/2008-w^‡^	Forage Triticale	NE 422T	67.0	October 17, 2007	--------^¶^
2008/2009-w	Winter wheat	Hatcher	67.0	September 8, 2008	July 21, 2009
2010-s	Proso millet	Huntsman	17.0	June 9, 2010	September 15, 2010
2011-s	Corn	Pioneer 37K11	30,000^§^ seeds	June 9, 2011	October 25, 2011
2012	Fallow	----	----	----	----
2012/2013-w	Winter wheat	Hatcher	67.0	September 26, 2012	July 21, 2013

^†^ Represents summer crops.

^‡^ Represents winter crops.

^§^ Corn seeding rate are in seeds ha^-1^.

^¶^ Grain were not available because forage triticale was harvested for livestock feed.

Two amendments: solid beef manure (M) and urea (46-0-0) as inorganic N fertilizer (F), each applied at two rates, low and high (Tables 2 and 3). The fertilizer was added annually, within a week before crop planting, for all the tillage treatments. The low, 33.6 kg N ha^-1^, fertilized rate (LF), was determined by estimating a conservative N requirement for typical dryland crops and applying that rate prior to planting each crop. The N rate for LF was 33.6 kg N ha^-1^ was used considering the productivity of this soil. The high, 67.2 kg N ha^-1^, fertilizer rate (HF) was twice the amount of the low rate.

**Table 2:** The amount of manure application (Mg ha^-1^) since the initiation of the study from 2006 to 2013 growing seasons. Manure was added close to the fall season anticipating the subsequence year crop except for 2012 where the manure was added in February of that year.

		---------------------------------- Beef manure^†^ ---------------------------------					
Manure application		Annually		Every other year (DT-2)^§^		Once every six years (DT-6)^¶^	
Month	Year	Low	High	Low	High	Low	High
		------------------------------ Mg Manure ha^-1^ ----------------------------------					
November	2006	11.7	36.5	22.4	64.3	64.3	188
October	2007	13.2	37.9	-------	-------	-------	-------
August	2008	10.3	29.8	19.7	57.1	-------	-------
November	2009	6.3	14.1	-------	-------	-------	-------
November	2010	8.7	26.2	16.1	50.4	-------	-------
February	2012^!^	5.4	12.1	-------	-------	-------	-------

^†^ Manure was applied close to the fall season of every year of application.

^§^ Represent manure application every other year (on even years).

^¶^ Represent manure application once every six years at study establishment estimating six years of crop N needs.

^!^ The manure application occurred in February on 2012.

**Table 3:** The amount of manure and chemical fertilizer application (kg N ha^-1^) since the initiation of the study from 2006 to 2013 growing seasons.

		----------------------- Beef manure^†^ -----------------------						Inorganic fertilizer^‡^	
Manure application		Annually		Every other year (DT-2)^§^		Once every six years (DT-6)^¶^		Annually	
Month	Year	Low	High	Low	High	Low	High	Low	High
		-------------------------- kg N ha^-1^ ---------------------------						--- kg N ha^-1^ ---	
November	2006	235	738	452	1299	1299	3390	33.6	67.2
October	2007	183	524	-------	-------	-------	-------	33.6	67.2
August	2008	112	323	214	620	-------	-------	33.6	67.2
November	2009	109	244	-------	-------	-------	-------	33.6	67.2
November	2010	124	372	229	716	-------	-------	33.6	67.2
February	2012^!^	81	181	-------	-------	-------	-------	33.6	67.2

^†^ Manure was applied close to the fall season of every year of application.

^‡^ Inorganic fertilizer was applied within a week before planting.

^§^ Represent manure application every other year (on even years).

^¶^ Represent manure application once every six years.

^!^ The manure application occurred in February on 2012.

Manure was added annually, in the fall or spring before tillage operations. For both NT and ST treatments, manure was added at a low rate (LM) that supplied the recommended N required for each crop. The assumption was made that only 25% of manure associated organic N would be available to the crop in the year of addition [21]. A high rate of manure (HM) was included in the study, where manure was supplied at three times the recommended N rate for the same crop in rotation. The DT-2 plots received manure biannually, at twice the annual rate of manure, to ensure enough N was supplied for two crops in rotation. Therefore, the LM rate was approximately twice the annual application for the recommended N rate for the crops in rotation while the HM rate was equivalent to six times the recommended N rate (twice the HM rate for the annual application). The DT-6 plots received manure once, just before the DT operation in fall of 2006 the total amount of manure added to the DT-6 was calculated to supply enough N for six consecutive dryland crops for the LM and the HM rate. The control (0N) treatment that received no manure or inorganic fertilizer was included with both NT and ST treatments, but was not included with the DT treatments.

In the effort of remediating this eroded site, a grass treatment and a grass mixed with legume treatments were included within the experimental design, to evaluate changes in soil nutrient status in a permanent vegetation system. The grass (Gr) treatment was a mixture of five native grass (Gr) species that were mixed at different rates as kg of Pure Live Seed per ha (kg PLS ha^-1^). The Gr mixture consisted of approximately 9 kg PLS ha^-1^ of western wheatgrass [*Agropyron smithii* (Rydb.)], 5.6 kg PLS ha^-1^ of green needle grass [*Nassella viridula* (Trin.)], 5 kg PLS ha^-1^ of sideoats grama [*Bouteloua curtipendula* (Michx.) Nash], 1.7 kg PLS ha^-1^ of blue grama [*Bouteloua gracilis* (H.B.K.)], and 4 kg PLS ha^-1^ little bluestem [*Schizachyrium scoparium* (Michx.) Nash]. The grass + legume (Gr + L) treatment contained the same amounts of Gr mixture (mention above) in addition to four other legume species. The legumes were mixed at equal amounts (0.28 kg ha^-1^) of American vetch [*Vicia americana*], purple prairie clover [*Dalea purpurea* (Vent.)], alfalfa [*Medicago sativa* (L.)], and cicer milkvetch [*Astragalus cicer* (L.)]. The legume percentage in the total Gr + L mixture represented approximately 4.2%. The Gr and Gr + L plots were planted to a forage sorghum cover crop in spring 2006 and the grass was planted in November 2006. The legume was mixed with the grass to provide nutrients necessary for grass establishment during the first year and the second of the grass growing season. Eventually, the grass will take over the entire plots. Over all, the combination of three tillage (NT, ST, and DT, three tillage frequencies (annual; DT-2, biannual; and DT-6, once every six years), three N amendments (0N, fertilizer, and manure), two N rates (L and H), and two grasses treatments (Gr and Gr + L) generated twenty different treatment combinations.

The beef manure or urea was broadcast and left on the surface in NT plots, incorporated with shallow tillage approximately 10 cm deep in ST plots, and incorporated with plowing, approximately 36 cm deep, in DT plots. Manure was chemically analyzed before each application for chemical characteristics (Olsen’s Agricultural Laboratory, Inc. McCook, NE) and N content evaluation (Table 4). Manure applications were made with the assumption that 100% of manure associated inorganic N (NH_4_^+^ and NO_3_^-^) content and 25% of manure organic N would be available through mineralization during the first season after addition [21]. Therefore, the annual manure applications throughout the 6-yr study period ranged between 5 to 13 Mg manure ha^-1^ y^-1^ for the low rate and approximately 12 to 38 Mg manure ha^-1^ y^-1^ for the high rate depending on fresh manure moisture content and inorganic N availability (Table 2). Fertilizer P, Mono-ammonium phosphate (11-52-0), was banded with the seed at planting of wheat, triticale, and millet on all plots (except Gr and Gr + L) at approximately 17 kg P_2_O_5_ ha^-1^.

**Table 4:** Chemical characteristic of the beef manure added to the research plots from 2006 to 2012^†^.

Year	Moisture	pH	EC	C:N ratio	Total N	Inorganic N^‡^	P_2_O_5_	K_2_O	Ca	Mg	Na	Cl	S	Zn	Fe	Mn	Cu
	%		dS m^-1^		----------------------------------- g kg^-1^ -----------------------------									---------- mg kg^-1^ ---------			
2006	36.6	6.2	15.5	------^§^	20.2	2.76	11.0	15.6	20.3	4.7	2.2	8.4	2.8	202	3622	170	34.3
2007	38.3	8.8	19.1	26.8	13.9	1.95	11.7	15.3	17.9	4.2	2.5	3.2	3.1	200	2387	126	34.3
2008	18.1	8.1	23.7	------	10.9	0.70	14.4	15.5	25.1	6.8	2.4	----	3.4	205	8300	200	0.0
2009	40.6	7.0	23.0	22.6	17.3	4.74	11.2	13.7	11.0	3.4	2.5	4.4	3.3	127	3184	131	31.3
2010	28.9	8.9	9.0	20.3	14.2	0.11	10.3	14.3	13.6	3.9	2.5	4.1	3.0	140	3450	125	30.1
2012	53.5	6.8	14.6	29.6	15.0	4.76	9.4	11.9	9.0	2.3	2.4	4.6	3.2	133	1346	100	24.6

^†^ Results are expressed on wet basis (as received).

^‡^ Inorganic N is the sum of NH_4_^+^-N and NO_3_^−^N content of manure.

^§^ Represents no available data.

### Soil sampling and analyses

Soil samples were collected in May of 2006 from each plot after the plot plan was laid out and before implementing the tillage and N treatments to determine the initial soil fertility conditions. Two sample cores, 3.2 cm dia., were taken and composited at 0–15, 15–30, and 30–60 cm depths from each plot using a hydraulic probe (Forestry Supplies, Inc. Jackson, MS). A third soil core was also taken and separated into the same depths to evaluate soil bulk density as described by [22]. In the spring of 2012, 6 years after the initiation of the study, soil samples from each plot were also collected using the same sampling protocols as used for 2006 sampling. The soil samples were taken between crop rows, avoiding the wheel-trafficked areas. Soil samples at all depths were then air-dried and ground to pass through a 2-mm screen as preparation for various soil chemical analyses.

Soil samples at 0–15 and 15–30 cm depths were analyzed for soil chemical properties by a commercial laboratory (Ward Laboratory, Kearney, NE). In short, electrical conductivity (EC) was analyzed with a 1:1 soil:water ratio using a glass electrode as reported by [23]. Soil acidity (pH) was analyzed with a 1:1 soil:water using a glass electrode ([24–25]. Soil phosphorus (P) was analyzed by the Olsen sodium bicarbonate method [26]. Soil extractable chemical potassium (K), sulfur (S), zinc (Zn), iron (Fe), manganese (Mn), cupper (Cu), calcium (Ca), magnesium (Mg), and sodium (Na) were extracted with neutral 1N NH_4_OAc as outlined by [27]. The extract was analyzed using an ICAP (Inductively Coupled Argon Cooled Plasma) Spectrometer (Thermo Fisher Scientific Inc., Waltham, MA). Soil samples from the 0–15, 15–30, and 30–60 cm depths were analyzed for total N and SOC. For soil total N, approximately 0.2 g of air-dried soil, was ground to a fine powder with a roller mill and evaluated by direct combustion (950°C) using CHN-2000 (LECO) Laboratory Equipment Corporation (Leco Co., St Joseph, MI). The SOC was evaluated by dry combustion methods outlined by [28]. Briefly, 6% sulfuric acid was added to a subsample (0.1 to 1.0 g) of a finely ground, air-dried soils to remove soil carbonate before performing a direct combustion at 950°C using a LECO CHN-2000. Sodium adsorption ratio (SAR) was calculated using the following formula: reported by [29]

$$SAR=[Na^{+}]/sqrt[(Ca^{2+}+Mg^{2+})/2]$$ (1)

Where SAR represents sodium adsorption ratio evaluated in millequivalents per liter (meq L^-1^); Na^+^, Ca^2+^, and Mg^2+^ represent water soluble sodium, calcium, and magnesium, respectively, measured in meq L^-1^.

### Statistical analysis

The treatment (consisting of combinations of tillage, N sources, and N rates combinations) and depth effects on soil chemical properties were tested using PROC MIXED procedure of SAS ver. 9.2 [30]. The F-test was performed by fitting a linear mixed model appropriate for a randomized complete block design. The effect of treatment on soil properties was tested as a fixed effect and the replication was considered random effects. The error term was equal to the residual after taking into account the effect of the replications. The depth was analyzed as sub-plots. The fixed effect was the depth and the depth interaction with treatment. The random effect was the replication and the replication x treatment interaction. The residual was used as the error term after taking into account the effect of replication and replication x treatment.

Changing of soil chemical properties influenced by treatments and study periods were evaluated through time. Soil properties influenced by time used the same PROC MIXED of SAS model that was used for depth evaluation. Soil properties influenced by time were also analyzed as split plot design similar to the depth analysis. The protected F-test was used to explain multiple comparisons of means using treatment differences. All results were considered significantly different at P < 0.05, unless otherwise noted.

## Results

### Soil chemical properties

In 2006, soil nutrient concentrations were similar for all areas of the field prior to establishment of the study. Significant treatment effects on change of soil nutrients were observed in 2012 after 6 years, except for Mn, in the 0–15 cm depth (Table 5) and Cu, Ca, and Mg in the 15–30 cm depth (Table 6). The addition of HM with both NT and ST significantly reduced soil pH and increased the other soil nutrients measured at 0–15 cm depth (Table 5) compared with the other treatment combinations. A significant reduction in soil pH was also observed in the 15–30 cm depth with the HM treatment associated with DT-2 and DT-6 treatments compared with other tillage treatment. Most other soil nutrients also increased with the HM treatment associated DT-2 and DT-6 tillages at the 15–30 cm depth (Table 6). Soil chemical properties associated with the grass and grass + legume plots, at any depth studied, were not statistically different than the control treatment (Tables 5 and 6).

**Table 5:** Soil chemical properties at 0–15 cm depth from spring of 2012 influenced by different treatments that included tillage treatments: No-tillage (NT); shallow tillage (ST), deep tillage every other year (DT-2); deep tillage once at the initiation of the experiment (DT -6); grass (Gr); and grass + legume (Gr + L) mixtures under different amendments: Chemical fertilizer at high rate (HF) and low rate (LF); beef manure at high rate (HM) and low rate (LM); no nitrogen added, control (0N).

Treatments				---------------------------------------------------- Soil chemical properties ---------------------------------------------------------											
Tillage	Nitrogen Type/rate	pH	EC	Olsen P	K	S	Zn	Fe	Mn	Cu	Ca		Mg	Na	SAR^†^
0–15 cm			ds m^-1^	----------------------------------------------------------- mg kg^-1^ ---------------------------------------------------------											meg L^-1^
NT	0N	7.90	0.36	13.6	634	8.25	0.65	3.75	3.23	0.46		3951	374.0	5.50	0.02
	HF	7.55	0.41	22.7	710	9.50	1.14	5.63	5.58	0.64		4151	361.8	9.50	0.04
	LF	7.33	0.35	16.0	674	9.75	0.71	6.08	6.48	0.66		3456	349.8	5.00	0.02
	HM	7.10	1.06	311.7	1264	45.25	10.36	8.15	8.28	1.45		3469	582.0	54.75	0.23
	LM	7.88	0.59	108.9	993	18.00	3.52	4.25	4.08	0.86		4674	409.5	34.75	0.13
ST	0N	7.90	0.36	19.0	703	7.75	0.69	3.75	4.65	0.56		4598	316.5	7.00	0.03
	HF	7.68	0.39	14.0	637	8.75	0.88	5.43	7.23	0.66		4398	357.3	6.50	0.03
	LF	7.88	0.37	13.0	651	8.00	0.69	3.33	3.65	0.49		3611	320.8	6.50	0.03
	HM	7.30	0.95	325.7	1331	35.00	10.15	8.23	5.88	1.38		3356	568.5	40.75	0.17
	LM	7.83	0.56	60.5	847	15.25	2.70	3.98	4.45	0.69		4285	368.8	25.00	0.10
DT-2	HF	7.98	0.43	15.7	571	10.25	0.85	3.78	2.90	0.63		5236	484.3	12.50	0.04
	LF	8.15	0.41	9.1	519	7.50	1.01	3.35	2.60	0.59		5397	457.0	6.75	0.02
	HM	7.95	0.77	65.5	761	16.75	2.78	3.88	3.55	0.71		5600	418.0	42.75	0.15
	LM	8.03	0.45	26.6	602	10.50	0.91	4.03	3.38	0.60		5123	428.5	13.50	0.05
DT-6	HF	8.03	0.38	15.3	602	7.75	0.53	3.43	3.50	0.48		4771	356.0	6.75	0.03
	LF	7.65	0.35	9.9	599	8.75	0.47	5.20	4.80	0.50		3752	440.3	7.25	0.03
	HM	8.13	0.48	22.4	830	8.75	0.63	3.00	2.85	0.51		5488	347.8	13.00	0.05
	LM	7.93	0.45	23.8	695	9.00	0.79	4.00	3.10	0.63		4737	544.5	8.75	0.03
Grasses	Gr	8.05	0.32	10.2	661	8.00	1.38	3.33	3.60	0.53		4770	340.0	11.25	0.04
	Gr + L	7.78	0.29	14.7	765	9.25	2.35	4.83	5.70	0.58		3476	390.0	6.75	0.03
		------------------------------------------------------------------------------------- P > F --------------------------------------------------------------------------													
	Treatment	0.0175	<0.0001	<0.0001	<0.0001	<0.0001	<0.0001	0.0202	0.2156	<0.0001		0.0074	<0.0001	<0.0001	<0.0001
	LSD_(0.05)_	0.57	0.157	62.8	139.1	7.11	2.18	2.999	3.95	0.25		1383	103.62	10.44	0.04

^†^ Represents Sodium Adsorption Ratio.

**Table 6:** Soil chemical properties at 15–30 cm depth from spring of 2012 influenced by different treatments that included tillage treatments: No-tillage (NT); shallow tillage (ST), deep tillage every other year (DT-2); deep tillage once at the initiation of the experiment (DT-6); grass (Gr); and grass + legume (Gr + L) mixtures under different amendments: Chemical fertilizer at high rate (HF) and low rate (LF); beef manure at high rate (HM) and low rate (LM); and no nitrogen added, control (0N).

Treatments				--------------------------------------------------- Soil chemical properties ------------------------------------------------------															
Tillage	Nitrogen Type/rate	pH	EC	Olsen P	K	S		Zn		Fe		Mn	Cu	Ca		Mg	Na		SAR^†^
15–30 cm			ds m^-1^	-------------------------------------------------------------- mg kg^-1^ ------------------------------------------------															meg L^-1^
NT	0N	8.23	0.42	5.55	313		6.75		1.01		3.23	3.25	0.55		5260	432.3		13.75	0.05
	HF	8.23	0.46	4.53	328		6.50		0.84		2.73	2.75	0.56		5376	523.3		10.50	0.04
	LF	8.25	0.45	4.38	346		7.00		1.12		3.73	2.95	0.67		5424	482.3		10.00	0.04
	HM	8.28	0.54	13.73	425		12.25		1.33		2.88	3.90	0.58		4993	392.0		34.75	0.13
	LM	8.30	0.50	5.58	304		8.25		0.89		2.63	3.00	0.49		5433	434.3		26.50	0.09
ST	0N	8.28	0.43	5.40	354		7.50		1.12		3.00	3.75	0.55		4828	455.8		9.75	0.04
	HF	8.30	0.38	4.90	281		6.25		0.94		2.85	2.88	0.54		5246	489.5		7.75	0.03
	LF	8.28	0.44	5.53	328		6.25		1.19		3.35	3.85	0.53		4688	405.5		13.00	0.05
	HM	8.25	0.57	7.33	374		12.75		1.09		2.95	3.43	0.60		5333	452.8		41.75	0.15
	LM	8.30	0.50	4.55	293		10.50		1.18		3.08	3.10	0.57		5360	436.5		26.25	0.09
DT-2	HF	8.13	0.67	7.80	425		8.25		0.76		3.25	4.73	0.56		5276	454.5		14.00	0.05
	LF	8.18	0.55	6.75	400		8.00		0.79		2.93	4.23	0.53		5299	409.5		7.50	0.03
	HM	7.93	1.22	54.05	732		44.75		2.62		4.10	7.80	0.71		5215	478.5		79.50	0.28
	LM	8.05	0.81	20.25	515		16.25		1.65		3.88	6.38	0.67		5279	481.8		33.75	0.12
DT-6	HF	8.23	0.45	6.90	352		7.00		1.01		3.05	4.43	0.55		4826	392.5		8.75	0.03
	LF	8.03	0.45	7.85	426		8.50		0.88		3.70	6.30	0.56		4185	419.3		8.50	0.03
	HM	8.03	0.86	54.18	653		42.25		3.59		5.40	8.63	0.99		5102	442.8		33.25	0.12
	LM	8.20	0.52	11.03	443		11.00		1.29		3.50	4.45	0.69		5179	475.8		22.50	0.08
Grasses	Gr	8.28	0.34	4.48	335		6.25		0.97		2.83	2.50	0.50		5412	467.3		7.75	0.03
	Gr + L	8.25	0.37	5.15	318		7.75		1.89		3.30	2.83	0.62		5249	478.5		11.25	0.04
		--------------------------------------------------------------------------------- P > F -----------------------------------------------------------------------																	
	Treatment	<0.0001	<0.0001	<0.0001	<0.0001		0.0042		0.0017		0.0385	<0.0001	0.3431		0.0783	0.4097		<0.0001	<0.0001
	LSD_(0.05)_	0.1407	0.19	18.60	111.86		19.99		1.19		1.33	2.55	0.41		693	97.75		12.68	0.045

^†^ Represents Sodium Adsorption Ratio.

The majority of the soil chemical properties tested were influenced by sampling time (years), treatment, and the interaction of time x treatments in the 0–15 and 15–30 cm depths (Table 7). At 0–30 cm depth, unlike the previous studies, the influence of time, treatment, and the interaction of time x treatment were statistically tested according to the tillage treatments where the DT-2 and DT-6 tillage treatments were analyzed independently of the NT, ST, and the grass treatments (Table 7). The relationship of time x treatment interaction at 0–30 cm depth associated with NT, ST, and the grass treatments was significant, similar to the 0–15 cm depth relationship, in 12 out of 13 measured elements except for Ca where the interaction was not significant. Unlike the DT-2 and DT-6 treatments, the significant interaction in time x treatment interaction at 0–30 cm depth was mixed between 0–15 and 15–30 cm depth. Ten out of 13 measured elements were significant, similar in their time x treatment interaction to the 0–15 cm except for pH, Cu, and Ca measurements where the interaction was not significant. However, 9 out of 13 elements were significant, similar to 15–30 cm except for pH, Fe, Mn, and Cu measurements where the interaction was not significant (Table 7). Averaged across treatment combinations, sampling depth greatly influenced the soil chemical constituents in the 0–15 cm compared with 15–30 cm depths (Table 8). In 2006, soil nutrients concentrations were greater in the surface 0–15 cm compared with the 15–30 cm depth. Higher soil pH in the 15–30 cm compared with 0–15 cm is related to high soil CaCO_3_ content at deeper depths in this study site. In 2012, soil chemical properties were significantly influenced by treatment combinations and depth studied (Table 8). Unlike 2006, the soil chemical properties in 2012 associated with DT-2 and DT-6 tillage treatments were analyzed independent of the NT, ST and the grass treatments. This analysis approach was followed in 2012 to evaluate the influence of years of deep tillage on soil chemical content in different depths. After six years (2012) of manure, fertilizer, and tillage treatments, the nutrient distribution between the two depths and the interaction between treatment and depth were influenced by tillage practices (Table 8). In 2012, soil chemical properties associated with NT, ST, and the grass treatments were different at different soil depths, except for Na and SAR measurements. The interaction between treatment and soil depth was significant except with four (pH, Fe, Mn, and Ca) out of 13 elements were the interaction was not significant (Table 8). Similar to 2006, the majority of nutrients associated with NT, ST, and the grass treatments were concentrated in the top 0–15 cm compared with 15–30 cm depth. However, the majority of nutrients with DT-2 and DT-6 tillage treatments were concentrated at the 15–30 cm compared with 0–15 cm depth and the treatment x depth interactions was insignificant except in four (Zn, Mg, Na, and SAR) out of 13 measured elements (Table 8).

**Table 7:** Statistical significance of the main and interaction effects of different treatment combinations and sampling time (years) on soil chemical properties at 0–15, 15–30, and 0–30 cm depth from 2006 to 2012 sampling dates.

Source of variation	pH	EC	Olsen P	K	S		Zn	Fe		Mn	Cu	Ca		Mg		Na	SAR^†^
0–15 cm		ds m^-1^	---------------------------------------------- mg kg^-1^ ------------------------------------------														meg L^-1^
Time (years)	***	***	***	***		***	***		ns^‡^	***	ns		**	***	***		***
Treatment	ns	***	***	***		***	***		*	ns	***		**	**	***		***
Time x Treatment	**	***	***	***		***	***		ns	ns	***		**	***	***		***
**15–30 cm**																	
Time (years)	**	***	***	***		**	***		**	**	ns		*	ns	***		***
Treatment	**	***	***	***		**	**		ns	**	ns		ns	ns	***		***
Time x Treatment	**	***	***	***		**	**		**	**	**		ns	**	***		***
**0–30 cm** **NT, ST, Grass**																	
Time (years)	***	***	***	***		***	***		**	***	ns		***	***	***		***
Treatment	ns	***	***	***		***	***		ns	ns	**		ns	ns	***		***
Time x Treatment	**	***	***	***		***	***		ns	ns	***		ns	**	***		***
**DT-2 and DT-6**																	
Time (years)	**	***	***	***		**	***		ns	**	ns		**	***	***		***
Treatment	ns	***	***	*		*	**		ns	ns	ns		*	ns	***		***
Time x Treatment	ns	***	***	***		**	**		ns	ns	ns		ns	*	***		***

^†^ Represent Sodium Adsorption Ratio

*** Significant at P < 0.0001.

** Significant at P < 0.05.

* Significant at P < 0.1.

^‡^ ns, not significant.

**Table 8:** Statistical significance of the effects of different treatments average across depths, sampling depth (cm) averaged across treatments, and the interaction of treatments x depth on soil chemical properties in 2006 and 2012 sampling dates.

Source of variation	pH	EC	Olsen P	K	S		Zn		Fe		Mn	Cu		Ca		Mg		Na	SAR^†^
2006		ds m^-1^	---------------------------------------------- mg kg^-1^ ------------------------------------------																meg L^-1^
Treatments	ns^‡^	ns	ns	ns		ns		ns		ns	ns	ns	ns		ns		ns		ns
Depth (cm)	***	***	***	***		***		*		***	***	***	***		***		**		ns
0–15 (mean)	8.0 b^¶^	0.41 a	8.5 a	561 a		8.7 a		0.5		4.3 a	10.0 a	0.7 a	4666 b		325 b		7.8 b		0.03
15–30 (mean)	8.3 a	0.35 b	2.4 b	334 b		6.3 b		0.4		3.1 b	3.4 b	0.6 b	5219 a		449 a		8.6 a		0.03
Treatment x Depth	ns	ns	ns	ns		ns		ns		ns	ns	ns	ns		ns		ns		ns
**2012** **NT, ST, Grass**																			
Treatments	ns	***	***	***		***		***		ns	ns	***	ns		ns		***		***
Depth (cm)	***	**	***	***		***		***		***	***	***	***		***		ns		ns
0–15 (mean)	7.8 b	0.50 a	77.5 a	822 a		15.2 a		2.9 a		5.1 a	5.2 a	0.7 a	4016 b		395 b		17.8		0.07
15–30 (mean)	8.3 a	0.45 b	5.9 b	333 b		8.2 b		1.1 b		3.0 b	3.2 b	0.6 b	5216 a		454 a		17.8		0.06
Treatment x Depth	ns	***	***	***		***		***		ns	ns	***	ns		***		**		**
**DT-2 and DT-6**																			
Treatments	ns	***	***	***		**		**		ns	ns	ns	**		ns		***		***
Depth (cm)	**	***	ns	***		**		**		ns	***	ns	ns		ns		***		***
0–15 (mean)	8.0 b	0.46 b	23.5 a	647 a		9.9 b		1.0 b		3.8 a	3.3 b	0.6 a	5013 a		435 a		13.9 b		0.05 b
15–30 (mean)	8.1 a	0.69 a	21.1 a	493 b		18.3 a		1.6 a		3.7 a	5.9 a	0.7 a	5045 a		444 a		26.0 a		0.09 a
Treatment x Depth	ns	ns	ns	ns		ns		**		ns	ns	ns	ns		**		**		**

^†^ Represent Sodium Adsorption Ratio

*** Significant at P < 0.0001.

** Significant at P < 0.05.

* Significant at P < 0.1.

^‡^ ns, not significant.

^¶^ The different lowercase letters within each column represents significant differences between depths.

**Fig 3:**
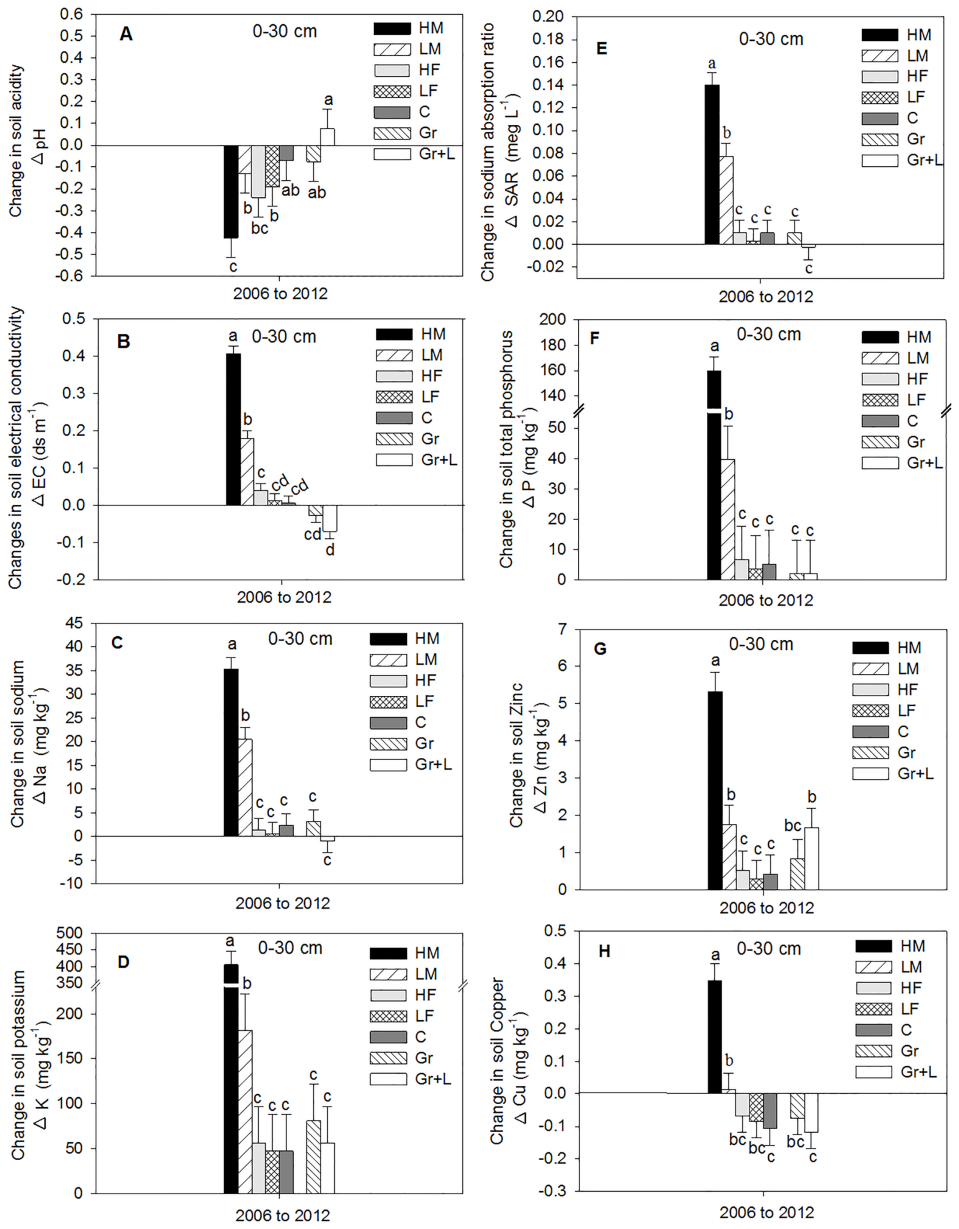
Change in soil chemical properties (A-H) from 2006 to 2012 at 0–30 cm depth averaged across tillage treatments as influenced by different N and grass legume mixtures treatments. HM treatment represents beef manure addition at three times the recommended N (high) rate; LM treatment represents beef manure addition at the recommended (low) rate; HF treatment represents chemical fertilizer (urea) addition at double the recommended N (high) rate; LF treatment represents inorganic fertilizer (urea) addition at the recommended (low) rate; C treatment represents no nitrogen addition (control); Gr represents the native grass; and Gr +L represents the mixture of native grass and legume. The error bars represent standard errors of the mean. The different lowercase letters represent significant differences among N treatments (P < 0.05).

The changes in soil nutrient concentration from 2006 to 2012 were averaged across NT and ST tillage treatments because soil nutrient concentration was not influenced by these two tillage treatments. Soil pH decreased in the 0–30 cm depth at the end of the six year period for all fertilizer treatments except for the Gr + L treatment (Fig 3A). The decrease in soil pH (from 2006 to 2012) was more pronounced with manure than with fertilizer and with high rate than with low rate of manure or fertilizer. The HM treatment decreased soil pH by approximately 0.295 units compared with LM treatment. Whereas, the HF treatment decreased soil pH by approximately 0.05 units compared with LF treatment (Fig 3A). The small decrease in soil pH from 2006 to 2012 associated with the 0N control treatments was probably due to field variability.

**Fig 4:**
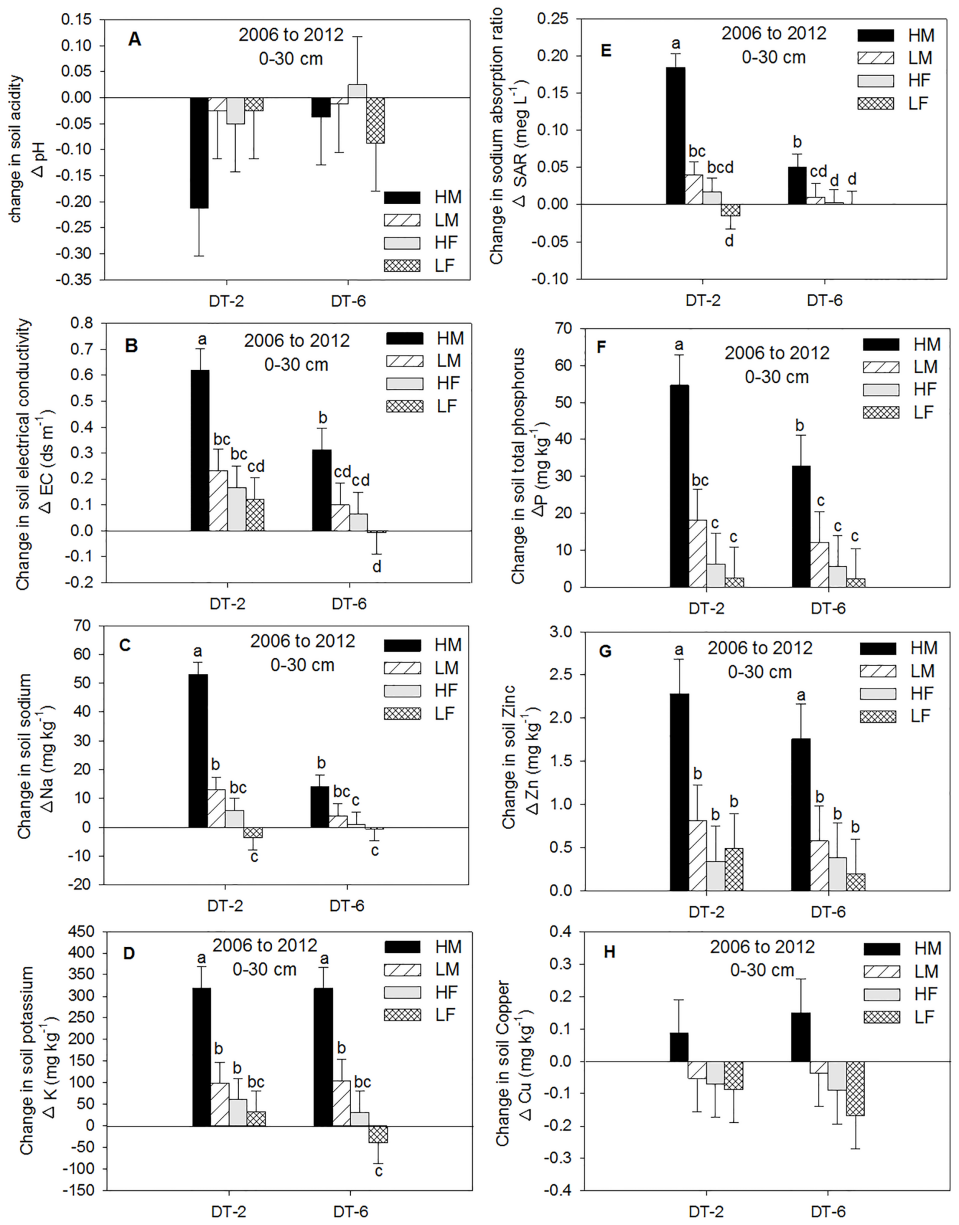
Change in soil chemical properties (A-H) from 2006 to 2012 at 0–30 cm depth as influenced by at different N and deep tillage (DT). DT-2 represents biannual deep tillage and manure (M) addition; DT-6 represents deep tillage and M addition once every six years; HM treatment represents beef manure addition at three times the recommended N (high) rate; LM treatment represents beef manure addition at the recommended (low) rate; HF treatment represents inorganic fertilizer (urea) addition at double the recommended N (high) rate; LF treatment represents inorganic fertilizer (urea) addition at the recommended (low) rate; C treatment represents no nitrogen addition (control). The error bars represent standard errors of the mean. The different lowercase letters represent significant differences among N treatments (P < 0.05).

The 2012 soil nutrients associated with control was considered the base line against which the other treatment combinations will be discussed accordingly. Subtracting the control treatment from different N rates and averaged across NT and ST, the pH was decreased by 0.4 units for HM, 0.17 units for HF, while the pH was decreased by 0.12 units for LF and by 0.06 units for LM in the 0–30 cm depth (Fig 3A). The soil pH associated with DT treatment decreased from 2006 to 2012 (Fig 4A) at 0–30 cm profile. After six years, the DT-2 associated with HM treatment reduced soil pH by approximately 5.6 fold and by 1.9 fold for LM treatment compared with DT-6 at the 0–30 cm depth.

Similar to soil pH, the changes in soil EC from 2006 to 2012 were also evaluated in relation to the control treatment. Annual HM additions significantly increased soil EC by approximately 2.3 fold compared with the LM treatment. This was expected because the HM application rate was 2.8 time higher than the LM rate with corresponding 2.4% average Na content. The high amount of Na associated with HM rate could contribute to increase soil EC compared with LM. With the inorganic fertilizer, Gr, and Gr + L treatments, soil EC was not different than the control treatment (Fig 3B). In 2012, the soil EC in the HM treatment was 2.3 fold higher (0.23 dS m^-1^) than the LM treatments and 11 fold higher (0.37 dS m^-1^) than the HF treatment. Soil EC with HF was 4.4 fold higher (0.03 dS m^-1^) compared with LF (Fig 3B).

The DT-2 treatment and manure addition frequency influenced soil EC changes from 2006 to 2012 in the 0–30 cm depth (Fig 4B). Three biannual applications of manure and DT (DT-2), increased soil EC with HM by approximately 2 fold (0.3 dS m^-1^) compared with DT-6, where the sum of 6 years of manure was added in a single application (Fig 4B). The HM and DT-6 treatment increased soil EC by approximately 3.1 fold (0.2 dS m^-1^) compared with LM and by 4.8 fold (0.15 dS m^-1^) compared with HF.

Averaged across tillage (NT and ST), changes in soil Na from 2006 to 2012 increased with HM by approximately 72% compared with LM where the fertilizer, Gr, and Gr + L were not different than the control treatment (Fig 3C). Higher amounts of soil Na was observed at the surface 15 cm with NT and ST while higher amounts of soil Na was observed at the 15–30 cm with the DT treatment (Tables 4 and 5). Similarly, changes in soil available K increased by approximately 2.2 fold with HM compared with LM where the other treatment combinations were not different than the control treatment (Fig 3D). The biannual HM application and DT (DT-2) significantly increased soil available Na by approximately 3.8 fold compared with HM and DT-6 (Fig 4C). Changes in soil available K associated with HM was significantly greater under both DT-2 and DT-6 than any other N treatments. However, one application of the sum of 6 years of manure (DT-6) and the distribution of approximately the same amount of manure throughout the study period (biannual application; DT-2) did not influence soil available K at 0- to 30 cm depth (Fig 4D).

**Fig 5:**
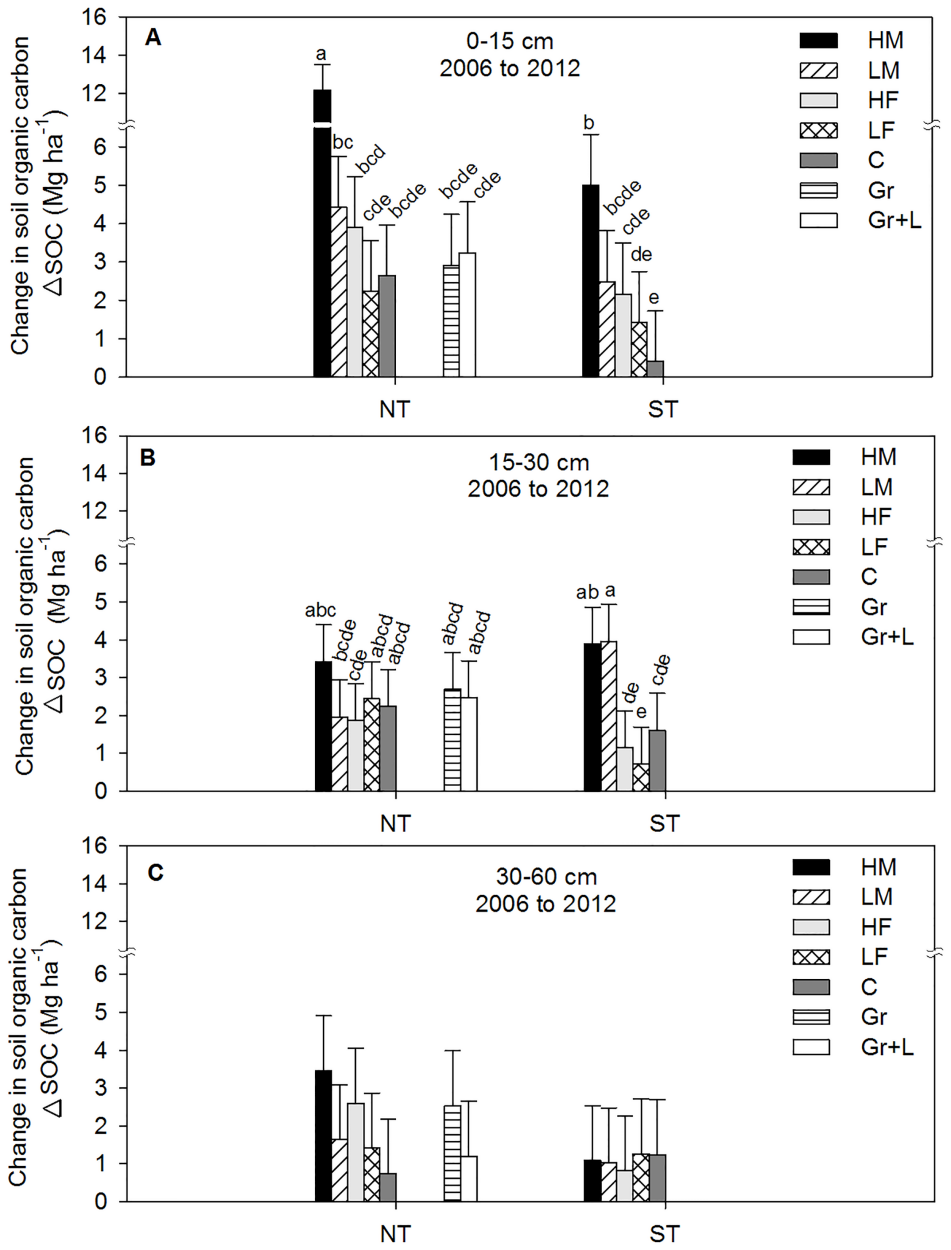
Change in soil organic carbon (ΔSOC) measured in Mg ha^-1^ from 2006 to 2012 as influenced by at different tillage treatments, soil depth, nitrogen (N) treatments, and grasses. NT represent no-tillage; ST represent shallow tillage; HM treatment represents beef manure addition at three times the recommended N (high) rate; LM treatment represents beef manure addition at the recommended (low) rate; HF treatment represents inorganic fertilizer (urea) addition at double the recommended N (high) rate; LF treatment represents inorganic fertilizer (urea) addition at the recommended (low) rate; C treatment represents no nitrogen addition (control); Gr represents the native grass; and Gr +L represents the mixture of native grass and legume. (A) represents 0–15 cm; (B) represents 15–30 cm; and (C) represents 30–60 cm. The error bars represent standard errors of the mean. The different lowercase letters represent significant differences among N treatments (P < 0.05).

One of the concerns of applying high amount of manure to agricultural land is increasing Na absorption ratio (SAR). The change in SAR at 0–30 cm depth was significantly greater with HM by approximately 80% compared with LM, but no differences were observed among F treatments, Gr, Gr + L, and control (Fig 3E). The changes in SAR associated with combinations of HM and DT-2 were greater than combinations of HM and DT-6 by approximately 3.7 fold (Fig 4E). Changes in soil microelements such as Zn and Cu were greater with HM than any other treatment (Figs 4E, 4H, 5G and 5H). Manure additions through time increased soil Zn and Cu concentration (Table 7). Higher concentrations of Zn and Cu, associated with NT, ST, and grasses treatments, were observed in the 0–15 cm depth than in the 15–30 cm depth. Nevertheless, Zn associated with DT-2 and DT-6 was grater at 15–30 cm than the 0–15 cm depth, but no differences in Cu was observed between depths (Table 8).

The change in soil Olsen (NaHCO_3_) extractable phosphorus (P), across tillage (NT and ST) was greater with HM than LM by approximately 4 fold. There were no differences in soil P observed among inorganic fertilizer treatments, Gr, Gr + L, and the control treatment (Fig 3F). Similar to the other chemical properties, the combination of HM and DT-2 contained approximately 66% higher amounts of soil P compared with the combination of HM and DT-6 (Fig 4F). Although P was added annually as P_2_O_5_ to the commercial fertilized plots, soil P increased tremendously with manure addition (Tables 5 and 6) at all depths studied. Higher amounts of extractable P were observed in the surface 15 cm with NT and ST, while higher amounts of extractable P were observed at the 15–30 cm with the DT treatment (Tables 5 and 6).

### Soil organic carbon and total nitrogen

Soil organic C (SOC) and soil total N (STN) were significantly influenced by time, treatment, and the interaction between the time and treatment at the 0–15 and 15–30 cm depths, but not at the 30–60 cm depth (Table 9). Changes in SOC, averaged across NT and ST, from 2006 (baseline) to 2012 in the 0–15 cm depth was highly influenced by treatment combination (P < 0.0001), depth studied (P < 0.0001), and the interaction between treatment x depth (P < 0.0002). Averaged across the treatments, ΔSOC was significantly greater at 0–15 cm than 15–30 cm, and greater at 15–30 cm than 30–60 cm (Fig 5A). Annual applications of HM combined with NT increased ΔSOC by approximately 2.4 fold (equivalent to 1.2 Mg C ha^-1^ yr^-1^) compared with HM associated with ST treatments in the 0–15 cm layer (Fig 5A). There were no difference in ΔSOC between inorganic fertilizer, control, and either grass treatments at all depth studied. The ΔSOC at 15–30 cm was significantly influenced by manure addition, especially with ST, (Fig 5B), but there were no differences observed at 30–60 cm depth (Fig 5C). After subtracting the control value from different treatments, the ΔSOC associated with HM increased by an average of 3.1 fold (1.4 Mg C ha^-1^ yr^-1^) for NT and by 2.3 fold (0.5 Mg C ha^-1^ yr^-1^) for ST, compared with HF treatment associated with each tillage in the 0–15 cm layer. The ΔSOC associated with LM and NT treatment increased by approximately 1.8 fold (0.3 Mg C ha^-1^ yr^-1^) compared with ST in the 0–15 cm depth. In the 0–30 cm depth, the ΔSOC with NT treatment was approximately 77% (3.7 Mg C ha^-1^ yr^-1^) greater with HM than LM. The ΔSOC with ST treatment was higher by 27% (0.4 Mg C ha^-1^ yr^-1^) for HM than LM (Fig 5).

**Table 9:** Statistical significance of the main and interaction effects of different treatment combinations and sampling time (years) on soil organic C (SOC) and total N (TN) at 0–15, 15–30, and 30–60 cm depth from 2006 to 2012 sampling dates.

Source of variation	Soil organic C (SOC)	Total N (TN)
	----------------------------------- Mg ha^-1^ --------------------------------	
**0–15 cm**		
Time (years)	< 0.0001	< 0.0001
2006 (mean)	15.6 b^¶^	1.8 b
2012 (mean)	17.9 a	2.1 a
Treatment	< 0.0001	< 0.0001
Time x Treatment	< 0.0001	< 0.0001
**15–30 cm**		
Time (years)	< 0.0001	< 0.0001
2006 (mean)	12.2 b	1.4 b
2012 (mean)	15.2 a	1.8 a
Treatment	0.0480	0.0078
Time x Treatment	0.0005	0.0009
**30–60 cm**		
Time (years)	0.0849	0.5543
2006 (mean)	17.1 a	1.9 a
2012 (mean)	17.9 a	1.9 a
Treatment	0.5610	0.4426
Time x Treatment	0.7231	0.2702

^¶^ The different lowercase letters within each column represents significant differences between depths.

**Fig 6:**
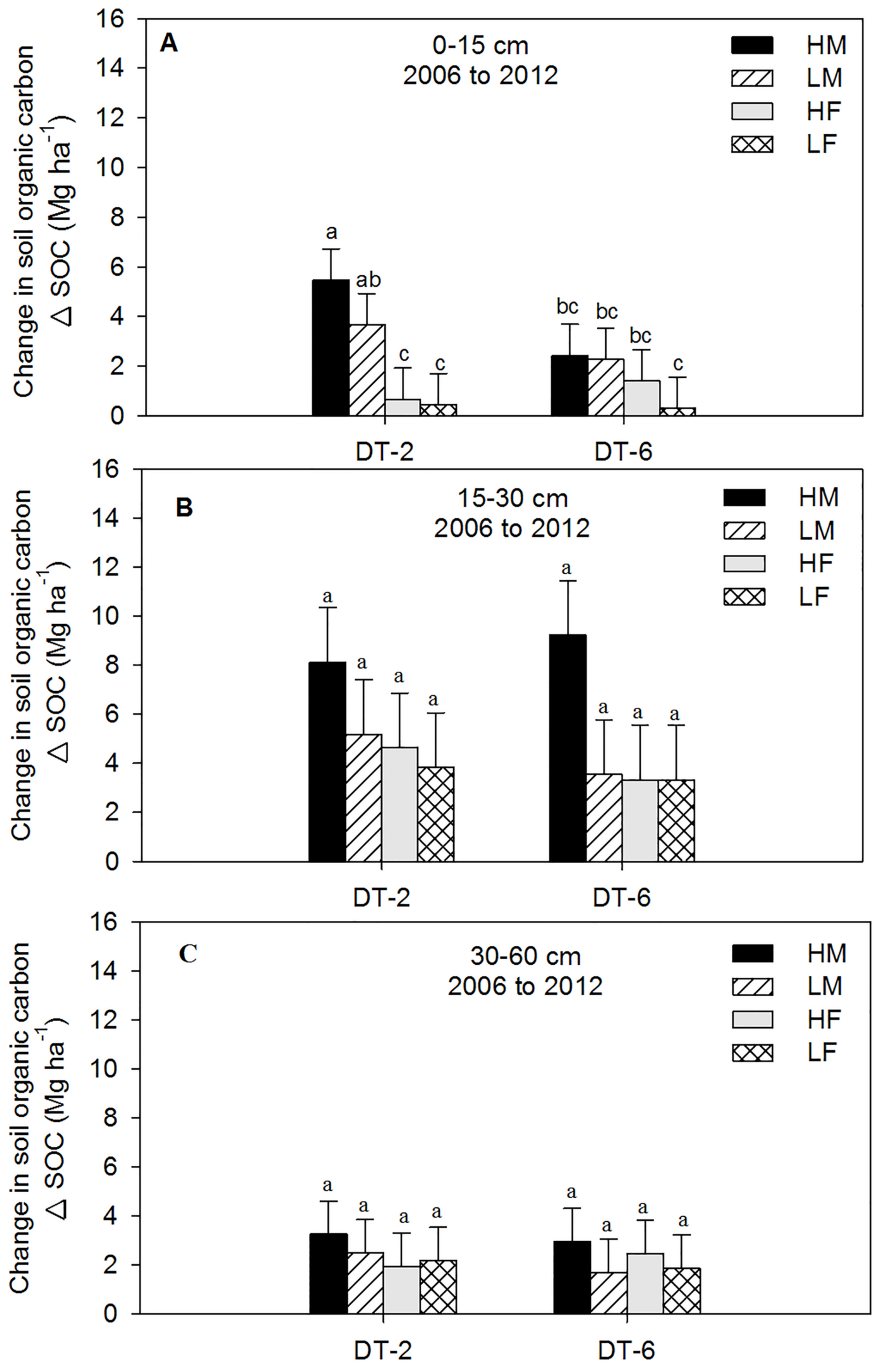
Change in soil organic carbon (ΔSOC) measured in Mg ha^-1^ from 2006 to 2012 as influenced by different N and deep tillage (DT). DT-2 represents biannual deep tillage and manure (M) addition; DT-6 represents deep tillage and M addition once every six years; HM treatment represents beef manure addition at three times the recommended N (high) rate; LM treatment represents beef manure addition at the recommended (low) rate; HF treatment represents inorganic fertilizer (urea) addition at double the recommended N (high) rate; LF treatment represents inorganic fertilizer (urea) addition at the recommended (low) rate; C treatment represents no nitrogen addition (control). (A) represents 0–15 cm; (B) represents 15–30 cm; and (C) represents 30–60 cm. The error bars represent standard errors of the mean. The different lowercase letters represent significant differences among N treatments (P < 0.05).

**Note:** Mention of commercial products and organization in this paper is solely to provide specific information. It does not constitute endorsement by USDA-ARS over other products and organization not mentioned. The US Department of Agriculture, Agricultural Research Service, is an equal opportunity/affirmative action employer and all agency services are available without discrimination.

The use of DT during this study significantly influenced ΔSOC. The ΔSOC, average across DT-2 and DT-6, was influenced by treatment combinations (P < 0.0133) and depth (P < 0.0001), but not by the interaction between treatment x depth (P < 0.3485). In the 0–15 cm depth, ΔSOC between 2006 and 2012 with DT-2 was approximately 33% greater with HM (equivalent to 0.3 Mg C ha^-1^ yr^-1^) compared with LM. Whereas the ΔSOC with LM associated with DT-2, was 4.5 fold (0.5 Mg C ha^-1^ yr^-1^) greater than HF (Fig 6A). There were no differences in ΔSOC observed among the treatments at 15–30 and 30–60 cm depth (Fig 6B and 6C). After six years, the ΔSOC associated with HM and DT-2 was approximately 55% greater (0.51 Mg C ha^-1^ yr^-1^) than HM associated with DT-6 in the surface 0–15 cm (Fig 6A). However at 15–30 cm, HM associated with DT-6 had a tendency to increase ΔSOC by approximately 12% (0.2 Mg C ha^-1^ yr^-1^) more than HM and DT-2 (Fig 6B). Similar to the NT and ST treatments, the ΔSOC in the 0–30 cm depth was greater with HM than LM by approximately 35% (0.77 Mg C ha^-1^ yr^-1^) for DT-2 and by 50% (0.97 Mg C ha^-1^ yr^-1^) for DT-6 treatment (Fig 6).

**Data Availability:** The authors confirm that all data underlying the findings are fully available upon request to the authors. All relevant data are within the paper.

**Experimental Site:** The research study site was carried on private crop land that is being rented from the owner by the Agricultural Research Service, Central Great Plain Research Station. The Agricultural Research Service, Central Great Plain Research Station have a permission (through the rental agreement) to conduct research and report the data. The field study is not involved with endangered or protected species.

## Discussion

### Soil chemical properties

The addition of HM greatly changed soil nutrient status by decreasing soil pH and increasing the amounts of soil nutrients. The depth where the changes in soil nutrients occurred depended on the tillage depth. For deep tillage treatments, the changes in soil nutrients were mainly observed at the lower depth studied (15–30 cm) compared with surface depth (0–15 cm) whereas, the changes with the majority of nutrients accrued at the surface 0–15 cm with NT and ST treatments. Manure was applied at the surface and then the soil was inverted by plowing, with the deep tillage treatment, resulting in the burial of the surface layer (soil + applied manure) to a depth of approximately 27–36 cm. In contrast, soil nutrient concentrations remained in the top 15 cm depth with the NT and ST treatments because of no disturbance with NT and the surface soil disturbance associated with ST at 8–12 cm depth. In different study locations, [12] also observed an increase in soil chemical concentrations at the surface 0–15 cm compared with the 15–30 cm depth with NT and ST practices. The low precipitation in this region and the calcareous nature of this soil may have promoted nutrient immobilization [19] and reduced soil nutrient movement below 15 cm with NT and ST treatments. Unlike the deep tillage treatments, where soil mechanical disturbance contributes to increased soil chemical concentrations by mixing manure and crop residue with the soil below 15 cm depth. These data also support our hypothesis that deep tillage redistributes soil nutrients to deeper depths compared with NT and ST.

The HM reduced soil pH by approximately 2 fold compared with HF, because the amount of N added with manure was calculated to be equivalent to three times the amount of N added with HF. In this study, the assumption that only 25% of manure associated organic N would be available to fulfill first year crop N needed as reported by [21], led to large amounts of total manure N added compared with the inorganic fertilizer were 100% of N addition assumed to be available for crop production. Therefore, the total amount of N added, in a given year, associated with HM ranged from 63% to 91% higher than HF and LM ranged from 59% to 86% higher than LF treatment (Table 3). The greater reduction in soil pH with HM was probably due to the additional amounts of N and C where the nitrification process (conversion from NH_4_^+^ to NO_3_^-^) that occurs under aerobic conditions results in the production of H^+^ ions and led to a decrease in soil pH [31–32]. Manure decomposition is likely to increase CO_2_ production that forms carbonic acid (H_2_CO_3_) with soil water. Upon dissociation of H_2_CO_3_ in soil, the H^+^ ions will form and also reduce soil pH [31]. The biannual manure addition associated with DT-2 could further contribute to reduction of soil pH compared with DT-6. The fresh manure rich with labile C and N fractions added every two years could contribute to the soil pH reduction compared with DT-6, where the labile C and N fractions could be less available for microbial activity and their denitrification and respiration processes. Overall, our results are consistent with previously reported research of decreasing soil pH with increasing N rates [12, 32–33] regardless to the tillage treatments.

Throughout the duration of this study (2006 to 2012), soil EC increased (Figs 3B and 4B) with increasing the rate of manure addition (LM *vs*. HM) and the frequency of manure addition (DT-2 *vs*. DT-6). Our data agrees with previous research that observed an increase in soil EC as the rates of N, either in the form of manure or ammoniacal fertilizer, increased ([12, 33–35]. The lower amount of soil EC associated with DT-6 was probably due to the fact that the entire amount of manure was added at once where the nutrients associated with EC level could be leached during the 6 year period of this study. Whereas, fresh manure with different EC level (Tables 2 and 4) was added biannually for the DT-2 treatment which could contribute to increase EC in DT-2 compared with DT-6. These data indicate that not only the amount of manure, but also the distribution of the same amount of manure throughout the study period can influence soil EC level. Using the 1.0 to 1.5 dS m^-1^ salt tolerant threshold sensitivity level for the majority of crops, grasses, and forages as reported by [36], our data indicates that the treatment combinations used in this study did not increase soil EC beyond the salt tolerant level that could affect crop production. However with HM treatment, we observed, the soil EC is approaching the salt tolerant threshold level specifically at the 0–15 cm depth with NT and ST (Table 5) and at the 15–30 cm depth with DT-2 (Table 6). In subsequent years, the EC level needs to be monitored closely and care needs to be taken when applying HM to avoid yield reduction due to high EC level.

The increase in soil EC observed in this study with HM addition could be related to the considerable amounts of available soil Na and K (Table 4; Fig 3C and 3D) and high amounts of manure-associated N (Table 1). The high amounts of N added with the HM treatment and the inorganic N generated from manure decomposition through time could contribute to increased soil EC in this treatment compared with other treatments. Our observation is supported by previous research [34, 37] that observed a positive relationship between soil EC and soil inorganic N, where EC was increased as soil inorganic N increased. Previously, [34] also reported that, in non-saline soils, the changes in soil EC is possibly related to the fluctuation in soil inorganic N, specifically NO_3_-N.

An analysis of our data demonstrates that high manure application rates increased SAR at the 0–30 cm depth regardless of manure application timing (annually, biannually, or once every 6 years). However, the SAR values were below 0.3 (Tables 5 and 6) which is far below the critical value of 15 where SAR could negatively affect soil physical properties, established by the [38]. Similarly, [12] also reported that five years of manure addition did not increase SAR beyond 0.5 on eroded land in western Kansas, USA.

The increase in soil extractable P through time was probably due to manure decomposition that converted manure-associated P to soil available P. Our findings agree with previous research that observed an increase in extractable soil P as the amount of manure applied increased [12, 17, 39]. Certainly, the DT treatments, that buried manure up to 30 cm deep, impacted the extractable P concentration in different depths compared with NT and ST. High accumulations of extractable P with HM compared with LM, under any tillage treatments, was related to the fact that manure application was based on the amount of N required for crop production. This observation agrees with previous research that documented the possibility of high accumulations of available P with high manure rate additions that is based on crop N needs [12, 39–40].

Previous research from the Eastern USA reported that high soil P accumulation due to manure additions can potentially cause surface water contamination through surface runoff, impairing water quality [17, 41]. Unlike the Eastern USA, our study was conducted in a semiarid region in the western USA on calcareous soils and low precipitation. The calcareous nature of our soil did not result in the high concentration of extractable P observed below the surface 0–15 cm depth, especially with NT and ST. The high concentration of extractable P at 15–30 cm associated with DT was likely related to manure being buried below the 15 cm depth rather than P leaching from the surface. Overall, low rainfall in this region and high soil pH reduces the risk of available P runoff and leaching to depths below 15 cm. Our data agreed with previous research that reported that calcareous soil may immobilize soil available P through P reactions with soil carbonates, which reduces P availability [39–40] and P movement down the soil profile [12]. Similar to soil P, the calcareous nature of the soil at this study site may have partially immobilized manure associated Zn and Cu at the surface thus preventing the movement of these elements below the 15 cm surface layer.

In this study, soil chemical properties in the grass and grass + legume treatments were not different than the control treatment. In the grass + legume treatment, the legume was used to provide nitrogen to support grass growth through N-fixation or through legume decomposition. It has been previously documented [42–43] that the advantages of legumes in grass-legume mixture such as atmospheric nitrogen fixation, reducing the need for fertilization by transferring the biologically fixed nitrogen to the grass in the mixture, support agro-ecosystem nutrient cycling, and reducing forage production. During the six year period of this study, the grass-legume mixture did not improve soil chemical properties compared with the grass alone and control treatments. The low precipitation associated with the central Great Plains Region, where the study is located, could have influenced the grasses and legume growth pattern. Previous research emphasized the importance of water availability for grass and legume growth, establishment, and biomass production where these parameters increased as soil water content increased [44–47]. Using data generated from 9500 sites within the central United States, [45] observed a grassland production gradient from east to west, where high annual precipitation in the east generated higher production compared with low annual precipitation and low production in the west. Since no inorganic fertilizer or manure was applied to the grass, grass + legume, and control treatments, the source of soil nutrients were from the decomposition of existing soil organic matter or the annually produced root and plant residues. However, the differences in changing soil chemical properties between the grasses and control treatments could be related to low plant residue, root, and soil organic matter decomposition associated with low precipitation in this region. Our observation agrees with previous research that observed a decrease in crop residue and soil organic matter decomposition with decreasing soil water content [48–51]. Therefore, it is likely that more than six years will be required to observe changes in soil chemical properties with the grass and grass + legume mixtures.

### Soil organic carbon and total nitrogen

After six years of different management practices, the analyses of these data show that the ΔSOC was mostly found at the surface 0–15 cm with NT and ST where the ΔSOC was mostly accumulated at 15–30 cm with DT. A similar pattern was observed with STN, but with different magnitude (data not shown). In this study, the distribution of SOC among the depth intervals was influenced by the depth of tillage between NT, ST, and DT practices. The ΔSOC, from 2006 to 2012, associated with NT treatments, averaged across HM and LM, was equivalent to 0.94 Mg C ha^-1^ yr^-1^ at 0–15 cm compared with 15–30 cm depth (Fig 5B). On the other hand, there were no differences in ΔSOC between depths with manure and ST treatments. This suggests that the ST treatment promoted manure decomposition and reduced SOC level compared with the NT treatment. A similar pattern was observed with STN, but with different magnitude. With the ST treatment the land is tilled at 8–10 cm depth with undercutter V-blade sweeps, which could enhance residue and manure decomposition and contribute to the lack of difference in ΔSOC between 0–15 and 15–30 cm depth. Our observation agreed with the previous research that indicates that tillage enhances soil organic matter decomposition by mixing plant residues or organic amendment into the soil, increasing aeration, exposing physically protected organic material for microbial degradation by disrupting soil aggregates, and enhancing dry-wet and freeze-thaw cycles [52–56]. No tillage, on the other hand, eliminates soil disturbance and allows soil organic matter accumulation [52, 56] and increases in SOC level.

The ΔSOC with DT treatment was mostly accrued at the 15–30 cm depth which was possibly related to the soil inversion that buries the surface soil containing manure and exposes the low soil C to subsurface soil. This observation agrees with [56], where they also observed the redistribution and stratification of SOC below the soil surface layer due to soil turnover with DT treatment. The high amount of ΔSOC at 0–30 cm depth with DT-6 than DT-2 is probably related to the large amount of manure that was added at the beginning of the study and buried compared with biannually manure addition with the DT-2 treatment. Previous research reported that the DT, especially moldboard plowing performed in this study, changed soil structure in the plowed layers because of the mechanical breakup of crop residues, the inversion and displacement of the surface soil by burying it into the sub-surface layer [56–59] and redistribute soil nutrients within the depth of tillage.

Regardless of tillage treatments, more positive changes in SOC were observed with HM than LM at 0–30 cm depth. The ΔSOC with the inorganic fertilizer treatments and the grass and grass+legume treatments were not different than the control treatment, indicating that either 6 years of the study are not sufficient to show the ΔSOC or that the biomass increase caused by inorganic fertilizer alone is not sufficient enough to increase SOC. The increase in ΔSOC associated with HM treatments was probably related to the annual addition of organic carbon associated with manure for the last six years. In a similar eroded site, [12] observed an increase in ΔSOC associated with HM compared with LM and F treatments. Other research [11] also reported an increase in SOC with manure addition on eroded land which was related to the immediate increase to the net primary production (root and shoot biomass as well as root exudates) that supported SOC formation.

## Conclusions

Six years of different manure application rates and tillage treatments on eroded cropland substantially increased soil chemical constituents compared with inorganic fertilizer and grass legume mixture treatments. The addition of manure, specifically at the high rates, increased plant available nutrient concentrations in the soil and decreased soil pH compared with other amendment types and rates. Manure applications, as a multi-nutrient source, improved soil nutrient status much more than could be expected from inorganic fertilizer alone. The type and the frequency of tillage also influenced the depth where the changes in soil chemical properties occurred. Our data indicates that after six years of study, changes in soil nutrient status associated with inorganic fertilizer and grasses treatments were not measured. Care needs to be taken when applying high manure rates to remediate eroded soils because high manure rates can increases soil EC to toxic levels. These data supported our hypothesis that the micronutrients supplied by manure improve overall soil nutrient status. The benefits of these treatments will be further evaluated for effects on soil quality and soil chemical properties in subsequent years.

## Abbreviations

Ca: calcium
Cu: Copper
DT-2: Deep tillage biannual
DT-6: Deep tillage once every six years
EC: electrical conductivity
F: inorganic fertilizer
Fe: Iron
HF: high rate of inorganic fertilizer
HM: high rate of beef manure
K: potassium
LF: low rate of inorganic fertilizer
LM: low rate of beef manure
M: beef manure
Mg: magnesium
Mn: manganese
Na: sodium
NT: no-tillage
P: phosphorus
pH: soil acidity
S: Sulfur
SAR: sodium adsorption ratio
SOC: soil organic carbon
ST: shallow tillage
STN: soil total nitrogen
Zn: Zinc
